# Anti-Cancer Effect of Neural Stem Cells Transfected with Carboxylesterase and sTRAIL Genes in Animals with Brain Lesions of Lung Cancer

**DOI:** 10.3390/ph16081156

**Published:** 2023-08-15

**Authors:** Jung Hak Kim, Jae Sung Ahn, Dong-Seok Lee, Seok Ho Hong, Hong J. Lee

**Affiliations:** 1 Research Institute, Humetacell Inc., Bucheon-si 14786, Republic of Korea; 2 Department of Neurosurgery, Asan Medical Center, University of Ulsan College of Medicine, Seoul 05505, Republic of Korea; 3 School of Life Sciences & Biotechnology, College of Natural Sciences, Kyungpook National University, Daegu 41566, Republic of Korea; 4 School of Life Sciences, BK21 FOUR KNU Creative BioResearch Group, Kyungpook National University, Daegu 41566, Republic of Korea; 5 College of Medicine and Medical Research Institute, Chungbuk National University, Cheongju-si 28644, Republic of Korea

**Keywords:** brain metastatic lung cancer, cell-based gene therapy, carboxylesterase (CE), secreted tumor necrosis factor-related apoptosis-inducing ligand (sTRAIL)

## Abstract

A metastatic brain tumor is the most common type of malignancy in the central nervous system, which is one of the leading causes of death in patients with lung cancer. The purpose of this study is to evaluate the efficacy of a novel treatment for metastatic brain tumors with lung cancer using neural stem cells (NSCs), which encode rabbit carboxylesterase (rCE) and the secretion form of tumor necrosis factor-related apoptosis-inducing ligand (sTRAIL). rCE and/or sTRAIL were transduced in immortalized human fetal NSCs, HB1.F3. The cytotoxic effects of the therapeutic cells on human lung cancer cells were evaluated in vitro with the ligands and decoy receptor expression for sTRAIL in the presence of CPT-11. Human NSCs encoding rCE (F3.CE and F3.CE.sTRAIL) significantly inhibited the growth of lung cancer cells in the presence of CPT-11 in vitro. Lung cancer cells were inoculated in immune-deficient mice, and therapeutic cells were transplanted systematically through intracardiac arterial injection and then treated with CPT-11. In resting state, DR4 expression in lung cancer cells and DcR1 in NSCs increased to 70% and 90% after CPT-11 addition, respectively. The volumes of the tumors in immune-deficient mice were reduced significantly in mice with F3.CE.sTRAIL transplantation and CPT-11 treatment. The survival was also significantly prolonged with treatment with F3.sTRAIL and F3.CE plus CPT-11 as well as F3.CE.sTRAIL plus CPT-11. NSCs transduced with rCE and sTRAIL genes showed a significant anti-cancer effect on brain metastatic lung cancer in vivo and in vitro, and the effect may be synergistic when rCE/CPT-11 and sTRAIL are combined. This stem-cell-based study using two therapeutic genes of different biological effects can be translatable to clinical application.

## 1. Introduction

Metastatic brain tumors are the most common type of intracranial neoplasm in adults and arise in 10–40% of cancer patients [1]. Brain metastasis is a main cause of cancer-related morbidity and mortality because it may rapidly devastate the function of the central nervous system (CNS) [2]. Non-small-cell lung cancer (NSCLC) accounts for about 85% of all lung cancer. It is primarily treated with surgical resection and is relatively insensitive to chemotherapy compared to small-cell carcinoma, another type of lung cancer, although chemotherapy has often been used either before or after surgery. Lung cancer, both small-cell-type and non-small-cell lung cancer (NSCLC), exhibits the highest occurrence of brain metastasis, with approximately 40% of lung cancer patients experiencing the development of brain lesions at some point throughout the disease progression [3]. While a single lesion of metastasis can be managed with local treatment such as surgery or radiosurgery, multiple lesions may need wider-range or systemic treatment, and the use of chemotherapy or whole-brain radiation has been a clinically plausible solution [4]. However, radiation therapy often results in unsatisfactory tumor control and can impair cognitive function. Chemotherapy is often insufficient, as the blood–brain barrier hinders the effective delivery of the therapeutic agents to the brain lesions, and moreover, it can cause unacceptable systemic toxicity [5]. To overcome the current limitation in the treatment of metastatic brain tumors, novel modalities have been suggested, one of which is neural stem cells (NSCs) as a vector to deliver therapeutic agents to brain lesions.

NSCs have been reported to possess inherent tumor-tropic properties as well as the characteristics of self-renewal, differentiation to multiple cell types, and migratory potential. In addition, recent studies have suggested that therapeutic strategies using the tumor tropism of NSCs are plausible in cancer treatment [6]. The use of NSCs as a delivery vehicle to treat disseminated intracranial lesions is highly attractive because therapeutic genes can be incorporated into NSCs, rendering them to target specifically invasive tumor cells [7]. Numerous studies have reported that such a strategy could be effective in animal models of malignant glioma [7,8,9] and also applicable in metastatic brain tumors from breast cancer, prostate cancer, and lung cancer [10,11,12]. NSCs carrying therapeutic suicide genes have been shown to be useful in treating primary or metastatic brain neoplasm [7,13,14]. Carboxylesterase (CE), which converts prodrug CPT-11 (irinotecan) to SN-38 (7-ethyl10- hydroxy-camptothecin), a potent topoisomerase inhibitor I, has been reported to be useful as a therapeutic agent. Moreover, it can be harbored in NSCs and attack tumor cells in primary or metastatic brain tumors [9,15,16]. In recent research, the effectiveness of using delivery therapeutic vector cells such as engineered monocytes, activated T cells, or stem cells, which are loaded with various therapeutic agents, has been reported in the treatment of malignant gliomas and metastatic brain tumors [17,18,19].

Tumor necrosis factor (TNF)-related apoptosis-inducing ligand (TRAIL, APO2 ligand or APO2L), a member of the TNF protein superfamily, is a promising chemotherapeutic agent since it damages and kills neoplastic cells but spares normal tissue [20,21]. TRAIL protein is expressed on the cell surface or present in a soluble form [22,23]. It activates the p53-independent extrinsic pathway by binding to the transmembrane death receptors DR4 (TRAIL receptor 1) and DR5 (TRAIL receptor 2). DcR1 (TRAIL receptor 3) and DcR2 (TRAIL receptor 3) also bind with TRAIL, but they lack a cytoplasmic death domain which turns on apoptotic downstream signals, thus acting as a decoy receptor [24].

TRAIL protein on the cell surface is cleaved by a specific metalloprotease and into soluble TRAIL (sTRAIL) to form a homotrimeric complex that has potent apoptosis-inducing activity [20,25]. Systemic administration of recombinant sTRAIL proteins has been reported to inhibit the growth of numerous tumor cells [21].

Since lung cancer is resistant to several chemotherapeutic agents, TRAIL receptor targeting therapy has been tried. However, a single use of TRAIL as an anti-cancer drug often produces insufficient clinical efficacy [26], and preclinical studies have shown that approximately half of the lung cancer cell lines are intrinsically resistant to the apoptotic activity of TRAIL receptor targeting therapy [27], including A549 cells. To overcome TRAIL resistance in tumor cells and enhance therapeutic efficacy, strategies to sensitize tumors for TRAIL-induced apoptosis have been suggested [28]. The combined use of different chemotherapeutic drugs or irradiation has been explored for this purpose and reported to considerably increase anti-tumor activity in preclinical studies [29,30]. CPT-11 has been reported to have a synergistic effect with TRAIL receptor targeting therapy [31,32].

In this investigation, the study involved the systemic transplantation of HB1.F3 human NSCs engineered with the genes for rCE and sTRAIL into animals bearing metastatic lung cancer in the brain. Subsequently, the administration of the prodrug CPT-11 was initiated. The main objective was to provide evidence supporting the notion that this approach effectively targets metastatic tumors.

## 2. Results

### 2.1. Establish Human NSCs Expressing rCE and sTRAIL

**Figure 1:**
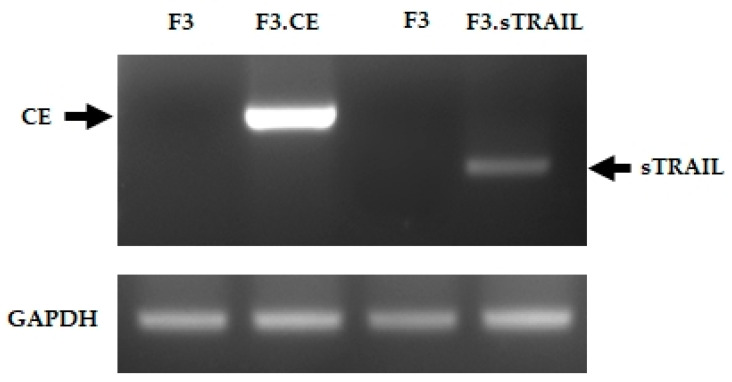
RT-PCR analysis of rCE and sTRAIL genes. RT-PCR analyses of F3 and F3.CE cells for expression of rCE gene. HB1.F3 (F3) human NSC line was transduced with a retroviral vector encoding the rabbit carboxyl esterase (rCE) gene. RT-PCR analyses of F3 and F3.sTRAIL cells for expression of sTRAIL gene. HB1.F3 (F3) human NSC line was transduced with a lentiviral vector encoding a secretable form of the human tumor necrosis factor-related apoptosis-inducing ligand (sTRAIL) gene.

**Figure 2:**
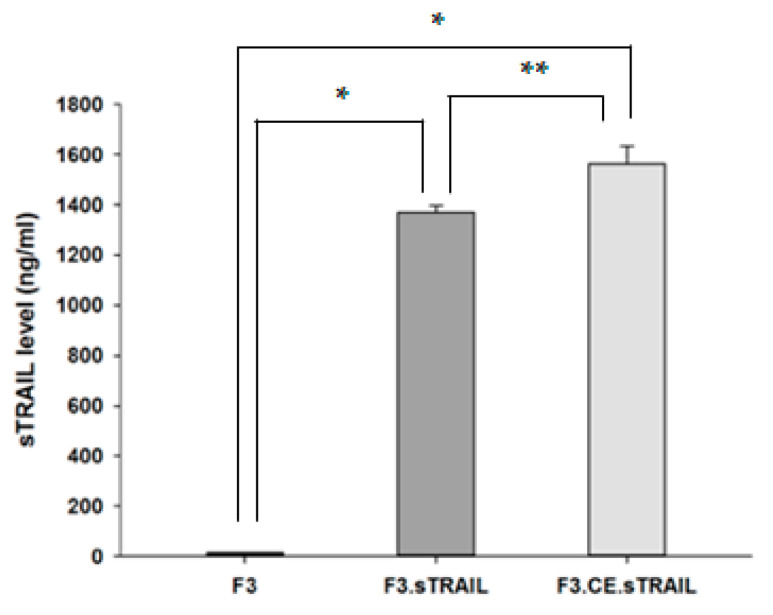
The secretion of sTRAIL from F3, F3.sTRAIL, and F3.CE.sTRAIL cells. The secretion of sTRAIL was measured using ELISA at 72 h after culturing cells. The amount of sTRAIL secreted from F3.sTRAIL and F3.CE.sTRAIL cells was >1300 pg/mL, whereas the amount of sTRAIL from parental F3 cells was <50 ng/mL. * *p* < 0.001; ** *p* < 0.05.

The expression of rCE and sTRAIL genes in the therapeutic human NSCs was demonstrated using RT-PCR. As expected, the rCE and sTRAIL transcripts were detected in F3.CE and F3.sTRAIL cells, respectively, but not in parental F3 cells (Figure 1). The secretion of sTRAIL was measured using ELISA in the cell media collected from F3, F3.sTRAIL, and F3.CE.sTRAIL cells (Figure 2). The amount of sTRAIL from F3.sTRAIL and F3.CE.sTRAIL was markedly higher (>1300 ng/mL) than that from parental F3 cells (<50 ng/mL).

### 2.2. Differential Cytotoxic Effects of CPT-11 on NSCs Expressing CE and sTRAIL

**Figure 3:**
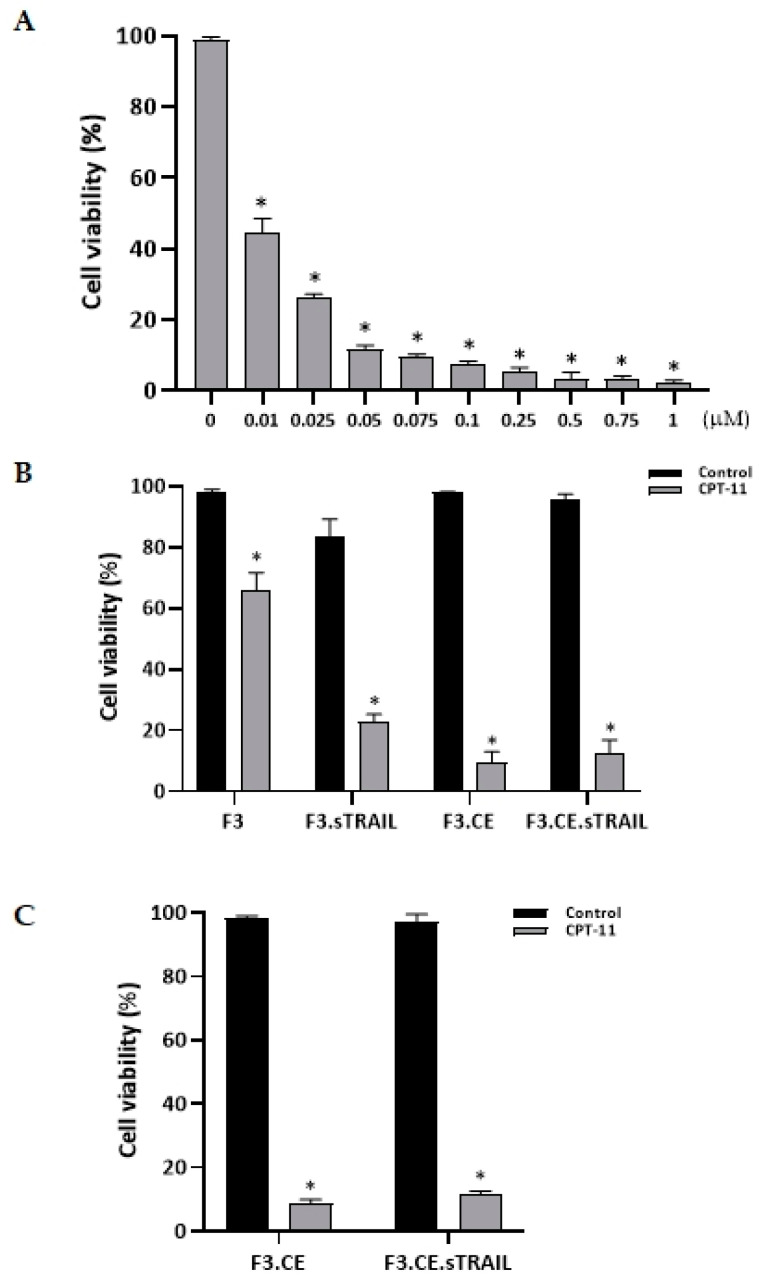
Cytotoxic effect of CPT-11 on therapeutic cells. (**A**) F3.CE cells were incubated with varying concentrations of prodrug CPT-11 for 48 h. CPT-11 at a concentration of 0.1 μM for 72 h killed >80% of F3.CE cells. (**B**,**C**). HB1.F3, F3.CE, F3.sTRAIL, and F3.CE.sTRAIL cells were incubated with or without 1 μM CPT-11 for 48 h. (**B**) Approx. 30% of F3 cells were killed by CPT-11, signifying a moderate cytotoxic effect of prodrug CPT-11. When F3.sTRAIL cells were exposed to the same amount of CPT-11, 70% of cells were killed, suggesting a probable additive cytotoxic effect. (**C**) F3.CE and F3.CE.sTRAIL cells were incubated with 1 μM CPT-11 for 48 h. Both F3.CE and F3.CE.sTRAIL cell were killed by >90%, signifying a strong cytotoxic effect of SN-38, converted from prodrug CPT-11 by the rCE enzyme expressed in the therapeutic cells. * *p* < 0.05.

To confirm the function of rCE, F3.CE cells were exposed to CPT-11 at concentrations ranging from 0.01 μM to 1 μM for 48 h. The survival of F3.CE cells was considerably reduced after 48 h of exposure to the prodrug CPT-11 at concentrations below 0.1 μM (Figure 3A). To confirm the supportive activity of rCE and sTRAIL toward CPT-11, the cytotoxic effect of CPT-11 on F3 or F3-based therapeutic cells was analyzed using a cell viability assay. F3 cells, parental NSCs, showed approximately 30% cell death after 48 h exposure to 1 μM CPT-11, whereas more cells (>70%) were dead in F3.sTRAIL cells with CPT-11, suggesting that the cytotoxicity of CPT-11 can be fortified by the presence of sTRAIL in the media. Moreover, the cytotoxic activity of CPT-11 with sTRAIL was markedly improved by rCE (>90%), signifying that the rCE enzyme in these cells efficiently converts prodrug CPT-11 to active topoisomerase inhibitor SN-38, resulting in a strong cytotoxic effect (Figure 3B).

### 2.3. In Vitro Bystander Effects on Lung Cancer Cells

**Figure 4:**
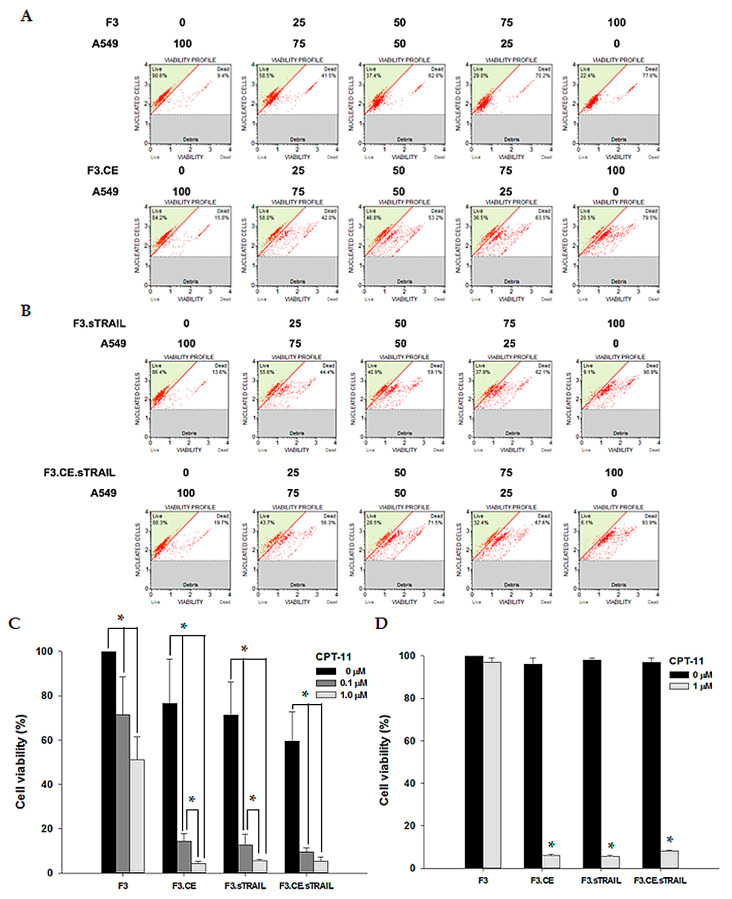
(**A**) A549 lung cancer cells were co-cultured with F3 or F3.CE cells in the presence of 1 µM CPT-11 for 48 h, and apoptotic cells and live cells were counted using a Muse^®^ Cell Analyzer (Millipore). When co-cultured with parental F3 NSCs, the apoptotic cell ratio was unaltered in any combination of cells. However, in A549 cells cultured with F3.CE cells in the presence of 1 µM CPT-11, the number of apoptotic cells dramatically increased after 48 h. In this condition, more cells were killed as the ratio of F3.CE cells to tumor cells increased. (**B**) A549 lung cancer cells were co-cultured with F3.sTRAIL or F3.CE.sTRAIL cells in the presence of 1 µM CPT-11 for 48 h, and apoptotic cells and live cells were counted. The apoptotic fraction of A549 cells increased when the cells were cultured with F3.sTRAIL or F3.CE.sTRIAL cells in the presence of 1 µM CPT-11 for 48 h. (**C**) Bystander killing effects of therapeutic cells were compared in a co-culture system with A549 cells. A549 cells with F3, F3.CE, F3.sTRAIL, or F3.CE.sTRAIL cells were seeded in 96-well plates (1 × 10^4^ total cells per well, A549 cells:therapeutic cells = 50:50). After 24 h, cells were treated with 0.1 or 1 µM CPT-11 for 48 h and cell survival was determined (each group, *n* = 3) (*p* < 0.05). Therapeutic cells expressing rCE, sTRAIL, or rCE plus sTRAIL exerted a cytotoxic effect on A549 cells in the presence of CPT-11, and more cells were damaged when a higher concentration of CPT-11 was added. (**D**) Bystander killing effects of therapeutic cells were compared in a co-culture system with H460 cells. H460 cells were plated with F3, F3.CE, F3.sTRAIL, or F3.CE.sTRAIL cells in 96-well plates (1 × 10^4^ total cells per well, H460 cells:therapeutic cells = 50:50). After 24 h, cells were treated with 1 µM CPT-11 for 48 h and cell survival was determined (each group, n = 3; *p* < 0.05). Therapeutic cells expressing rCE, sTRAIL, and rCE plus sTRAIL exerted a cytotoxic effect on H460 cells when the cells were treated with CPT-11. * *p* < 0.05.

The bystander effects of the therapeutic cells on A549 and H460 cells were assessed using a co-culture system. When A549 lung cancer cells were treated with 1 μM CPT-11 in the absence of therapeutic cells or in the presence of parental F3 cells, there was little effect on cell survival until 48 h after treatment. However, when A549 cells were co-cultured with F3.CE cells (with varying ratios of tumor cells to therapeutic cells: 100:0, 75:25, 50:50, or 25:75), their survival was significantly reduced after 48 h of exposure to 1 μM CPT-11 (Figure 4A). Cell survival after co-culture of A549 with F3.sTRAIL or F3.CE sTRAIL cells was also measured (Figure 4B). Interestingly, co-culture with F3.sTRAIL also resulted in increased cell death in the presence of CPT-11, and the ratio of apoptotic versus live cells was comparable to those of co-culture findings with F3.CE or F3.CE.sTRAIL. Thus, these bystander effects were compared among co-cultures of A549 cells with F3, F3.CE, F3.sTRAIL, or F3.CE.sTRAIL cells (tumor cells: therapeutic cells = 50:50) with an addition of 0, 0.1, or 1 μM CPT-11. Cell survival in A549 was reduced in co-culture with F3.CE or F3.CE.sTRAIL after treatment with CPT-11 and also in co-culture with F3.sTRAIL (Figure 4C), as depicted above. The same results were obtained with the H460 cell (Figure 4D). Without CPT-11, co-culture with therapeutic cells had no effect on the survival of both tumor cells, except for co-culture with F3.sTRAIL or F3.CE.sTRAIL, which leads to mild cytotoxic effects (Figure 4B,C), which can be attributed to the influence of secreted sTRAIL from these cells.

### 2.4. Changes in the Expression of TRAIL Receptors

**Figure 5:**
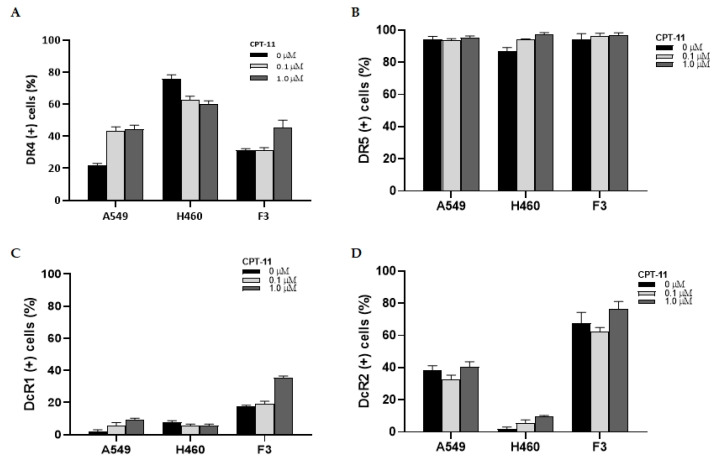
(**A**) The rate of DR4 receptor expression was high in H460 cells (>70%) but relatively low in A549 and F3 NSCs (<30%) when measured with FACS analysis. After 0.1 or 1 µM CPT-11 treatment for 24 h, the rate of DR4 expression of A549 and F3 NSCs was increased, whereas that of H460 cells was largely unaltered or slightly decreased. (**B**) The rate of DR5 receptor expression was similarly high (>80%) in both lung cancer cells and F3 NSCs. Changes after CPT-11 treatment were not evident in any cells. (**C**) The rate of DcR1 expression was relatively low in all cells examined, <5% in A549 and H460 tumor cells and approximately 13% in F3 NSCs. Exposure to CPT-11 did not affect the expression rate of DcR1 in the cells, except in F3 NSCs, in which DcR1 expression moderately increased with 1 µM CPT-11 treatment. (**D**) The rate of DcR2 expression was 40% in A549 cells and 71% in F3 NSCs. In H460 cells, DcR1 expression was low (<5%). CPT-11 did not alter the expression of DcR1.

FACS analysis of TRAIL receptor DR4, DR5, DcR1, and DcR2 in lung cancer tumor cells and F3 NSCs revealed different rates of expression and response patterns to CPT-11 treatment. When DR4 expression was measured, >70% of H460 cells were found to have DR receptors on their surface, whereas the DR4 expression rate of A549 and HB1.F3 cells was less than 30% (Figure 5A). This may be related to the higher sensitivity of H460 cells to TRAIL therapy. After 0.1 or 1 μM CPT-11 was added to the cell culture, the change in DR4 expression rate was measured, and the expression in A549 and F3 cells increased by approximately 70%. The increased expression was noted with a low-concentration (0.1 μM) treatment of CPT-11 in A549 cells without changes in cell morphology related to cellular damage or survival. On the contrary, the expression rate of DR4 in H460 cells was not definite or slightly decreased when exposed to CPT-11. When the expression of DR5 was analyzed, all the cells examined showed a high rate of expression (>80%), and this expression was not changed after low- and high-concentration CPT-11 treatment (Figure 5B). The expression of DcR1 in tumor cells was low (<5%) in both A549 and H460 cells, and the exposure to CPT-11 did not change the rate. In F3 cells, DcR1 receptor expression was mildly higher than in tumor cells (13%) and increased after the addition of 1 μM CPT-11 by approximately 90% (Figure 5C). DcR2 was considerably expressed in A549 cells (40%) and in F3 NSCs (71%) but was found to be low in H460 cells. The presence of CPT-11 in cell culture did not change the expression of DcR2 in all cells (Figure 5D). From this finding, A549 cells showed less DR4 and more DcR2 receptors, whereas H460 cells showed more DR4 and less DcR2 receptors. The treatment of CPT-11 increased DR4 expression in A549 cells, which may be related to previous reports on the TRAIL resistance of A549 cells [27] and CPT-11 synergism to TRAIL apoptosis [33,34].

### 2.5. In Vivo Therapeutic Efficacy

**Figure 6:**
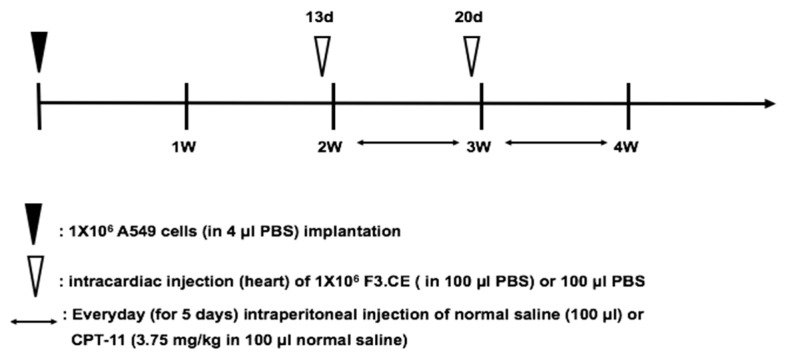
Timeline for the establishment of the lung cancer brain metastasis animal model and subsequent treatment with therapeutic cells and CPT-11. A549 human non-small-cell lung adenocarcinoma cells (1 × 10^6^ cells in 4 µL PBS) were implanted into the brains of 7-week-old SCID mice (n = 9). Thirteen and twenty days after tumor cell implantation, therapeutic cells (1 × 10^6^ cells in 100 µL PBS) were intracardially injected, and CPT-11 (10 µM, 3.75 mg/kg) was intraperitoneally injected for a further 5 days (for a total of 10 days). The mice were divided into six groups as follows: in group 1 (control group), the mice were treated with 100 µL PBS without CPT-11 as a negative control; in group 2 (F3.CE group), the mice were treated with F3.CE cells in 100 µL PBS (intracardiac injection) without CPT-11; in group 3 (CPT-11 group), the mice were treated with CPT-11 alone; in group 4 (F3.sTRAIL group), the mice were treated with F3.sTRAIL; in group 5 (F3.CE/CPT-11 group), the mice were treated with F3.CE cells and CPT-11; and in group 6 (F3.CE.sTRAIL/CPT-11 group), the mice were treated with F3.CE.sTRAIL cells and CPT-11. Two days following the last CPT-11 injection, all mice were euthanized, and their brains were processed for histology.

The timeline for the establishment of the lung cancer brain metastasis animal model and subsequent treatment using therapeutic cells and CPT-11 is shown in Figure 6.

Thirteen and twenty days after stereotaxic A549 cancer cell implantation into the brains of immune-deficient mice, animals were subjected to intracardiac injection of saline (control and CPT-11 group), F3.CE cells (F3.CE and F3.CE/CPT-11 group), F3.sTRAIL cells (F3.sTRAIL group), or F3.CE.sTRAIL cells (F3.CE.sTRAIL/CPT-11) group). Forty-eight hours after the cell injection, the control, F3.CE, and F3.sTRAIL groups received intraperitoneal injections of normal saline, whereas the other groups received intraperitoneal injections of CPT-11 (3.75 mg/kg) every day for 5 days after each cell injection (total 10 days).

**Figure 7:**
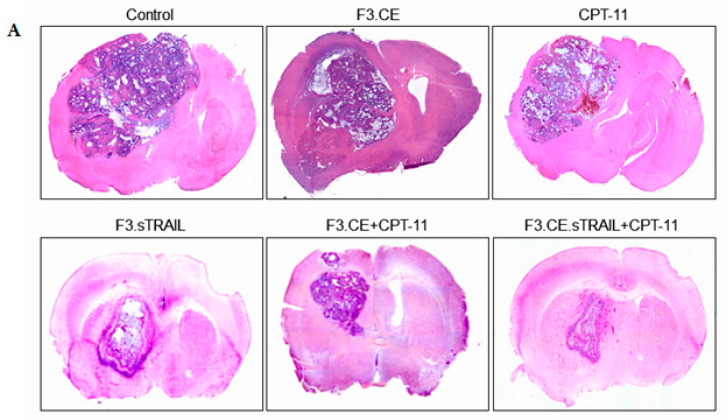

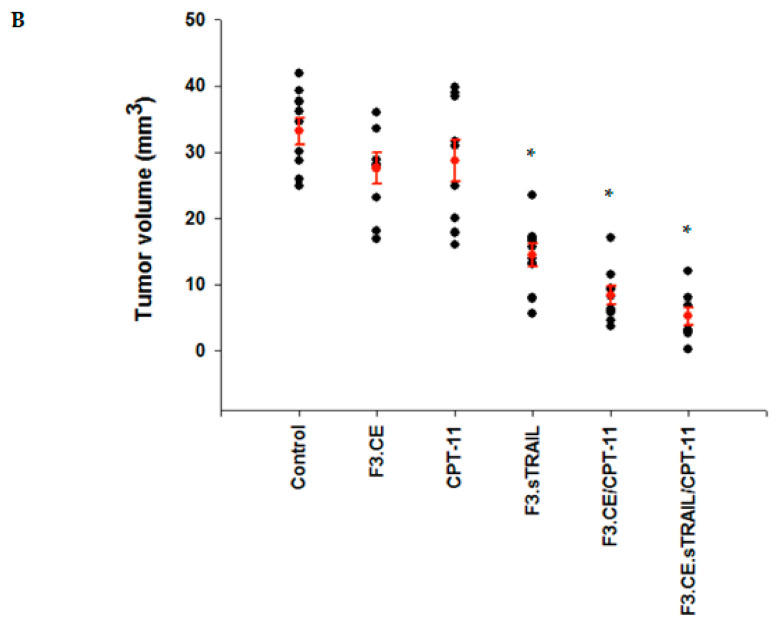
(**A**) Representative images of brain slices from each group. Tumor-bearing mice treated with F3.CE cells plus CPT-11, F3.sTRAIL cells, and F3.CE.sTRAIL cells plus CPT-11 showed significantly smaller tumor volumes than other groups including the sham control group, tumor-bearing animals transplanted with F3.CE cells but without CPT-11 treatment, and animals injected with CPT-11 but without therapeutic cell transplantation. (**B**) Treatment with therapeutic cells and CPT-11 has a significant therapeutic effect in vivo. Tumor-bearing mice treated with F3.sTRAIL cells, F3.CE cells plus CPT-11, and F3.CE.sTRAIL cells plus CPT-11 showed significantly smaller tumor volumes than other groups, including the sham control group, tumor-bearing animals transplanted with F3.CE cells but without CPT-11 treatment, and animals injected with CPT-11 but without therapeutic cell transplantation (*p* < 0.05). The tumor volume of the F3.CE.sTRAIL/CPT-11 group was significantly smaller than that of the F3.sTRAIL group or the F3.CE.sTRAIL group. * *p* < 0.05.

**Figure 8:**
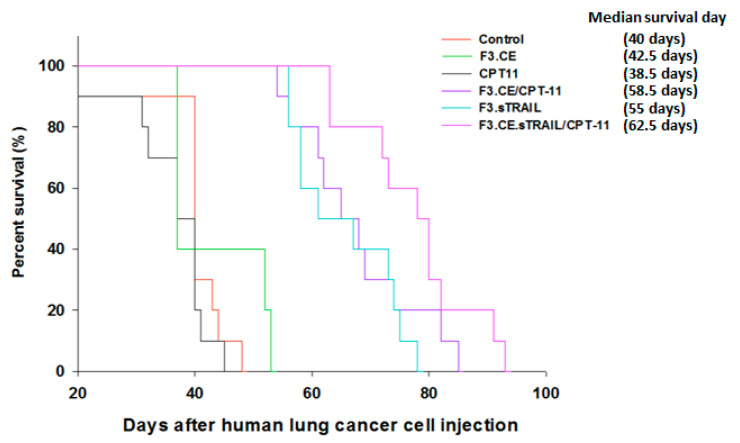

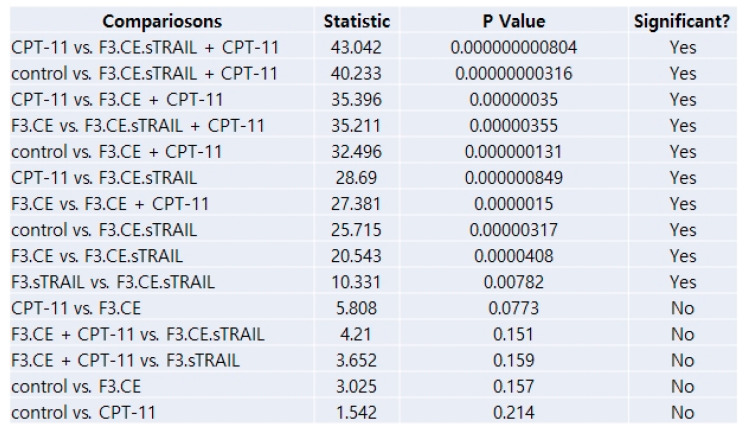
Survival analysis of mice treated with the therapeutic cells and CPT-11. The Kaplan–Meier graph shows significantly longer survival periods of the mice administered with the F3.sTRAIL, F3.CE/CPT-11, and F3.CE.sTRAIL/CPT-11 cells than those of the control, F3.CE group, or CPT-11 group (*p* < 0.05). The survival of the F3.CE.sTRAIL/CPT-11 group was the longest, significantly longer than that of F3.sTRAIL, but the difference from that of the F3.CE/CPT-11 group was not statistically significant (*p* = 0.151).

Brain sections prepared from animals treated with F3.sTRAIL, F3.CE plus CPT-11, and F3.CE.sTRAIL plus CPT-11 showed a significant reduction in tumor volume. Large tumors were observed in brain sections of control animals and animals treated only with F3.CE cells without subsequent CPT-11. The in vivo therapeutic efficacy of F3.CE cells against lung cancer brain metastasis was determined with tumor volume measurement (Figure 7) and survival analysis (Figure 8).

When tumor volumes were determined in brain tissue 2 days after the last CPT-11 injection, the brains of the mice in the F3.CE/CPT-11 group showed significantly reduced tumor volumes (median ± s.d = 8.43 ± 1.37 mm^3^) compared with the control (33.2 ± 2.02 mm^3^), F3.CE (27.6 ± 2.36 mm^3^). The mice of the CPT-11 alone group harbored similar-sized tumors (28.7 ± 3.13 mm^3^) to the control and F3.CE group. The tumor volume of the F3.sTRAIL group was also significantly smaller (14.5 ± 1.76 mm^3^) than those of the control, F3.CE, and CPT-11 alone group. The tumor volume of the F3.CE.sTRAIL/CPT-11 group showed significantly smaller (5.27 ± 1.23 mm^3^) tumor volume than that of the control and was also significantly different from those of the F3.CE/CPT-11 or F3.sTRAIL groups. There was an 84.1% reduction in tumor volume in the F3.CE.sTRAIL/CPT-11 group compared with the control group.

## 3. Discussion

In the present study, we have demonstrated that NSCs expressing therapeutic genes such as rCE or sTRAIL exhibit anti-cancer effects on metastatic brain tumors originating from lung cancer. Furthermore, the combination of rCE/CPT-11 and sTRAIL therapy shows synergistic anti-cancer effects. The stem-cell-based findings show promise for potential translation into clinical studies, as indicated by an ongoing investigational phase I and II clinical trial involving adult patients with recurrent glioblastoma (clinical trial ID NCI-2022-10170; http://clinicaltrials.gov/ct2/show/NCI-2022-10170 (accessed on 5 May 2023)).

NSCs encoding sTRAIL may have cytotoxic activity on tumor cells by binding TRAIL receptors on the cell surface. TRAIL-targeted therapy has been suggested to be a useful anti-cancer treatment because TRAIL selectively affects tumor cells without interfering with normal tissue [35]. Tumor cells abundantly express TRAIL-containing death domain, such as DR4 and DR5, while non-neoplastic cells have decoy receptors that are deficient in structures for the transmission of a death signal to the downstream cascade [26]. However, several tumor cells have been reported to express insufficient TRAIL sensitivity and may be resistant to TRAIL therapy, which could be partly attributed to different receptor expressions among various cancer cells. A549 and H460 cells, which were utilized in this study for in vitro and in vivo experiments, have been known to have different susceptibilities to TRAIL apoptosis [27]. We also confirmed that DR4 was intensively expressed in H460 cells, not in A549 or NSCs. For TRAIL-resistant cancer cells such as A549, combination chemotherapy could be a useful method, as exposure to a chemotherapeutic agent might render cancer cells more susceptible to TRAIL apoptosis [34]. When receptor expression was examined after CPT-11 treatment for 24 h, DR4 expression in A549 increased by 70%, and decoy receptor DcR in the present study might partially explain augmented anti-tumor effects in in vitro and in vivo experiments.

A combination of rCE/CPT-11 therapy with the TRAIL strategy may also provide safety benefits, as well as treatment efficiency [36]. There have been concerns that long-term habitation of foreign cells inside the brain, where immune rejection is relatively privileged, might result in hazardous events, such as secondary malignancy or over- or unwanted production of biologically active materials. NSCs that can produce sTRAIL alone could not eliminate themselves since non-neoplastic cells are resistant to TRAIL apoptosis. However, cell death could be readily induced when NSCs additionally express the rCE gene, or other types of suicide gene, by prescribing a prodrug that can be converted to a highly cytotoxic agent [37]. This property may allow efficient and safer cell-based treatment.

The utilization of NSCs as drug delivery vehicles presents a potentially innovative approach to address the limitations of conventional treatments by enabling the targeted delivery of high concentrations of chemotherapeutic agents specifically to the site of the lesion. This localized delivery strategy holds the potential to overcome the drawbacks associated with current chemotherapy, such as low efficacy and significant systemic toxicity. Recent research has highlighted the advantages of genetically engineered NSCs, suggesting their potential utility in gene therapy for the treatment of brain tumors [7,13]. The use of HB1.F3 immortalized human fetal NSCs in intracranial primary or metastatic brain tumors has been published in multiple studies. F3 human NSCs engineered to express Escherichia coli cytosine deaminase (CD), which can convert prodrug 5-fluorocytosine (5-FC) to active 5-fluorouracil (5-FU), reduced brain tumor growth in vitro and in vivo [11,12,38,39,40,41,42,43].

F3 NSCs encoding the rCE gene also demonstrated migratory potential to the brain tumor site and anti-tumor effects on subdural medulloblastoma [8] and metastatic tumors from breast cancer [9,10] after implantation. Carboxyl esterase (CE) is responsible for the conversion of prodrug CPT-11 (irinotecan) into its active metabolite SN-38, which is a topoisomerase I inhibitor that exhibits a 1000 times greater potency. In this study, a gene for rabbit CE was utilized as the activity of human carboxylase to convert prodrug CPT-11 is known to be lower, and rabbit CE has been more efficient [38,44].

CPT-11 is a chemotherapeutic agent that specifically kills dividing cells. CPT-11 is approved for numerous cancers, including malignant gliomas, as it can penetrate the blood–brain barrier (BBB) with relatively high efficiency. Promising results have been demonstrated in preclinical studies [8,9,10,13], and also, a clinical phase II study is under investigation in patients with malignant brain tumors [45,46]. However, the main obstacle that limits CPT-11 from more active clinical application in primary and metastatic brain tumors is systemic toxicity, including severe gastrointestinal disturbance, interference in immune function and leukopenia, and hepatotoxicity [46,47,48]. Considering that a higher dosage of drugs is prescribed in patients with intracranial lesions, balancing the efficacy with the adverse toxicity is crucial to accomplish optimal treatment results. Currently, this issue has been addressed by combining additional chemotherapeutic agents, such as bevacizumab, which can synergistically enhance anti-tumor activity by targeting different biological mechanisms involved in tumor growth inhibition [47].

The treatment strategy described in this study also could potentially be used to treat other types of common metastatic cancer in the brain, such as breast cancer and melanoma. Additionally, this treatment strategy could be used in combination with other cancer therapies, such as radiation therapy and immunotherapy, considering multimodal strategies for metastatic brain tumors.

Another effective method is to localize drugs in the target region without raising the systemic concentration, such as convection drug delivery and the use of biological vectors containing therapeutic material.

We inoculated the human NSCLC cell lines into the brains of the immune-deficient mice to investigate the therapeutic efficacy in vivo. This type of metastatic brain tumor model cannot fully reproduce the biological characteristics of cancer metastasis as it bypasses the early stages of the metastatic cascades of spearing from the primary sites and vascular invasion. While other types of metastatic models, such as the systemic inoculation of cancer cells, spontaneous spreading from the orthotropic injection, or a genetically engineered mouse model, may well recapitulate the actual clinical process and be more suitable for studying the physiological processes of metastasis or validating preventive methods, they are not easy to use for analyzing treatment effects, mainly because the size of the lesions varies, and many animals reach the humane endpoint due to extracranial cancer burden before brain lesions are formed. Especially in the case of NSCLC, the available models are very limited. Despite numerous drawbacks, xenograft models have the advantage of analyzing and comparing treatment effects using lesions of relatively uniform size, making them widely used for NSCLC treatment analysis.

The present study provides evidence that the brain transplantation of human NSCs, encoding the genes of the suicide enzyme rCE and sTRAIL, combined with the systemic administration of CPT-11 could be regarded as an effective therapeutic approach for treating metastatic brain tumors originating from lung cancer. This strategy could potentially find valuable applications in patients with brain metastases, offering valuable insights to inform the development of future metastasis therapeutics. While the therapeutic scheme suggested and demonstrated in this study shows promising results for the treatment of brain metastatic lung cancer using NSCs encoding rCE and sTRAIL genes, one limitation is that the xenograft animal models for metastatic brain tumors may not fully replicate the conditions found in human disease, including the tumor microenvironment and immune responses. Therefore, the intended therapeutic mechanism may differ in actual clinical settings. Another potential weakness is the relatively small number of animals used in the study, although significant beneficial results were obtained with nine animals in each group. Despite these limitations, we aim to conduct more robust and well-designed studies using various animal models and well-thought-out approaches.

## 4. Materials and Methods

### 4.1. Cell Culture

HB1.F3 (F3) is a stably immortalized human NSC cell line derived from human fetal telencephalon at 15 weeks of gestation. The derivation was accomplished by introducing a retroviral vector encoding v-myc [49,50,51,52]. F3 cells, including F3.CE (encoding rabbit CE), F3.sTRAIL (encoding sTRAIL), and F3.CE.sTRAIL (encoding both), were cultured in Dulbecco’s modified Eagle’s medium (DMEM) supplemented with 10% fetal bovine serum (FBS, Invitrogen, Grand Island, NY, USA), 2 mM L-glutamine, 100 units/mL penicillin, and 100 mg/mL streptomycin (DMEM-10% FBS). The A549 and H460 human non-small-cell lung adenocarcinoma cell lines were purchased from the Korean Cell Line Bank (Seoul, Korea) and American Type Culture Collection (ATCC) and maintained in DMEM-10% FBS.

### 4.2. Genetic Engineering of Therapeutic Cells

The clonal F3.CE, F3.sTRAIL, and F3.CE.sTRAIL human NSC lines were derived from the parental F3 NSC line. For transduction and establishment of stable cell lines encoding rabbit CE, an expression plasmid with rabbit CE was constructed using the retroviral pIRESneo vector (Clontech, Palo Alto, CA, USA) [38,49,53] and transduced with MV12 envelope-coding plasmid cDNA into pA317 cells. Parental HB1.F3 cells were infected with retrovirus collected from the supernatant of packaging cell cultures, and clonal cells were selected with neomycin for 7 days.

For the transduction of sTRAIL genes, a lentivirus containing the secretion form of the TRAIL gene (sTRAIL) was engineered [54]. The recombinant replication-deficient lentiviral vector encoding the gene for enhanced green fluorescent protein was constructed using pLHCX.eGFP (Clontech, Palo Alto, CA, USA) [49], and transduction was performed as described above. Transduced cells were selected by fluorescence-activated cell sorting (FACS) using GFP expression [55].

### 4.3. Reverse Transcription–Polymerase Chain Reaction (RT-PCR)

For RT-PCR, cells are lysed by Trizol, and total RNA was isolated using an RNA isolation kit, according to the manufacturer’s protocol (Promega, Beijing, China). One μg of total RNA was reverse-transcribed into first-strand cDNA using oligo-dT primer and AMV reverse transcriptase (Takara, Shiga, Japan). The cDNA was amplified using 30 PCR cycles.

Successful transduction of therapeutic genes of rCE and sTRAIL in the cells was confirmed with the reverse transcriptase–polymerase chain reaction (RT-PCR) using the primer pairs for rCE and sTRAIL genes (Table 1). Expression of topoisomerase and TRAIL in lung cancer cells A549 and H460 was also confirmed using primer pairs for topoisomerase genes (TOP1, TOP2a, TOP2b, TOP3a, TOP3b, TOPBP1) and sTRAIL genes (DR4, DR5, DcR1, DcR2). 

### 4.4. Enzyme-Linked Immunosorbent Assay (ELISA)

To measure the concentration of sTRAIL in cell media, 1 × 10^6^ cells of F3, F3sTRAIL, and F3.CE.sTRAIL were plated in a 6-well plate and cultured for 72 h. Cultured media were used to measure sTRAIL concentration using a TRAIL ELISA kit (Abcam, Cambridge, UK). 

To measure sTRAIL concentration in mice brains, 1–2 mm brain tissue with a tumor region was collected and homogenized in lysis buffer using Tissuelyse (Qiagen, Hilden, Germany). The supernatant was used for ELISA as described by the manufacturer.

### 4.5. Cell Viability Assay

To confirm the activity of rCE in therapeutic cells, the cytotoxic effects of CPT-11 on F3, F3.CE, F3.sTRAIL, or F3.CE.sTRAIL cells were analyzed using a cell viability assay. For each cell line, cells (1 × 10^4^ per well) were planted in 96-well plates. Twenty-four hours after seeding, 0.5–10 μM of CPT-11 (Hanmi Pharmaceutical, Seoul, Korea) was applied for 48 h, the status of the cells was analyzed using a microscope, and viability and apoptosis were determined with a colorimetric assay (Cell Counting Kit-8; Dojindo Molecular, Gaithersburg, MD, USA) and Muse^®^ Cell Analyzer (Millipore, Billerica, MA, USA).

### 4.6. In Vitro Bystander Effect Experiments

A549 and H460 human non-small-cell lung adenocarcinoma cells were plated in 96-well plates with F3 or F3.CE, F3.sTRAIL, or F3.CE.sTRAIL cells. The ratios of A549 or H460 cells to F3 or F3.CE or F3.sTRAIL or F3.CE.sTRAIL were 75:25, 50:50, 25:75, or 0:100. The cells were maintained in DMEM-10% FBS. After 24 h of cell growth, 1 μM CPT-11 was added to the mixed cell cultures. After 48 h, cell viability was determined as described above.

### 4.7. FACS Analysis of TRAIL Receptors

Surface expression of TRAIL receptors, DR4, DR5, DcR1, and DcR2, was determined with fluorescence-activated cell sorting (FACS). A549 and H460 lung cancer cells and parental F3 NSCs were plated and incubated in the presence of 0, 0.1, or 1 μM CPT-11 for 24 h. After dissociation, cells were labeled with DR4, DR5, DcR1, and DcR2 antibodies conjugated with phycoerythrin (PE) (1:3.5 dilution) for 30 min, as described in the manufacturer’s manual. After washing with PBS, the labeled cells were detected using FACS aria II (BD Biosciences; San Jose, CA, USA). Anti-DR4 (TRAIL R1), anti-DR5 (TRAIL R2), anti-DcR1 (TRAIL R3), and anti-DcR2 (TRAIL R4) antibodies were purchased from R&D systems (Minneapolis, MN, USA).

### 4.8. Animal Model of Lung Cancer Brain Metastasis

The animal experiments in this study have been reviewed and approved by the Animal Care and Use Committee of Chung-Ang University (Certification CA11-0086). For direct tumor cell implantation into the brain, SCID mice (7 weeks old) were anesthetized and secured in a rodent stereotaxic frame. A small drill hole was made at 2 mm to the left and 1 mm anterior to the bregma. Then, 1 × 10^6^ A549 human lung cancer cells in 4 μL of phosphate-buffered saline (PBS) were injected into the brain using a Hamilton syringe at a depth of 2 mm (anterior/posterior (AP) + 1.0 mm, medial/lateral (ML) + 1.7 mm, dorsal/ventral (DV) − 3.2 mm) over a period of 10 min.

### 4.9. In Vivo Therapeutic Efficacy

The therapeutic efficacy of each cell line was evaluated in six study groups (control, F3.CE, CPT-11, F3.sTRAIL, F3.CE/CPT-11, F3.CE.sTRAIL/CPT-11), containing 9 mice per group. Thirteen and twenty days after A549 cell injection, animals were subjected to intracardiac arterial injection of 100 μL of PBS (control or CPT-11 group) or 1 × 10^6^ therapeutic cells (F3.CE, F3.sTRAIL, F3.CE/CPT-11, and F3.CE.sTRAIL/CPT-11 groups); forty-eight hours after the cell injection, the control and the F3.CE and F3.sTRAIL groups received intraperitoneal injection of normal saline (100 μL), whereas the CPT-11, F3.CE/CPT-11, and F3.CE.sTRAIL/CPT-11 groups received CPT-11 (3.75 mg/kg in 100 μL normal saline) every day for 5 days after each cell injection, a total of 10 days.

Two days after the last injection, the brains were removed and cut into 4–6 mm thick coronal slices. For tumor volume measurement (largest width^2^ × largest length × 0.5), the brain slices were fixed in 10% formalin in phosphate-buffered saline (PBS), embedded in paraffin, sectioned into 4 μm sections using a microtome, and stained with H&E.

For survival analysis, seven mice were included in each group and treated as described. They were monitored for the presence of symptomatic intracranial mass, indicated by signs such as incoordination, lethargy, or weight loss exceeding 25% of the maximal body weight. When these symptoms became evident during observation, it was considered as mortality, and the respective animal was euthanized.

### 4.10. Statistics

Data from each experiment are presented as the mean ± standard deviation (SD). Statistical comparisons between groups were conducted using the Student’s *t*-test. One-way ANOVA was utilized to compare the means of each group in the in vivo study. Survival analysis was performed using the Kaplan–Meier and log-rank tests. *p* values < 0.05 and <0.001 were considered statistically significant.

## 5. Conclusions

The present research offers proof that transplanting human NSCs into the brain, which carry the genes for the suicide enzyme rCE and sTRAIL, along with the systemic administration of CPT-11, could be considered an effective treatment approach for metastatic brain tumors derived from lung cancer. In this study, we have demonstrated that NSCs expressing therapeutic genes like rCE or sTRAIL display anti-cancer properties against metastatic brain tumors originating from lung cancer. Additionally, the combination of rCE/CPT-11 and sTRAIL therapy exhibits synergistic anti-cancer effects. These findings utilizing stem cells hold promise for clinical application, as supported by an ongoing phase I clinical trial in adult patients with recurrent glioblastoma. This strategy may be readily applicable to patients with brain metastases and can serve as a guide for the development of future treatments for metastatic tumors.

## Figures and Tables

**Table 1:** Primer pairs for RT-PCR to confirm encoded gene expression.

Gene	Sense	Antisense
DR4	GCGGGGAGGATTGAACCAC	CGACGACAAACTTGAAGGTCTT
DR5	ATGGAACAACGGGGACAGAAC	CTGCTGGGGAGCTAGGTCT
DcR1	ACCAACGCTTCCAACAATGAA	CTAGGGCACCTGCTACACTTC
DcR2	GTTGGCTTTTCATGTCGGAAGA	CCCAGGAACTCGTGAAGGAC
Top1	CCAGACGGAAGCTCGGAAAC	GTCCAGGAGGCTCTATCTTGAA
Top2a	ACCATTGCAGCCTGTAAATGA	GGGCGGAGCAAAATATGTTCC
Top2b	GGTTCGTGTAGAGGGGTCAAG	GCCGTCCACCTTTTGTAGTTG
Top3a	CCCGAAGACCGTGCCTTTT	CTCATGCGACCGTTTGACAG
Top3b	TGGCGAGAAGACCGTGTTC	AATCACCGTATTTCCCCTGGA
Topbp1	TGAGTGTGCCAAGAGATGGAA	TGCTTCTGGTCTAGGTTCTGT
Top1mt	ACGAAGACGGGGTGAAGTG	CCGGAAAACCTCCTTTGTTGTG
rCE	ATGATGGCCTGGCTCTTTCT	TCTCGGAAAATTGCTCGATG
sTRAIL	GGAATCATCAAGGAGTGGGC	TGGACCATTTGTTTGTCGTT
GADPH	CATGACCACAGTCCATGCCATCACT	TGAGGTCCACCACCCTGTTGCTGTA

## Data Availability

Data is contained within the article.
